# Supplementary material for: Clinical Utility and Validation of the Krakow DCM Risk Score—A Prognostic Model Dedicated to Dilated Cardiomyopathy
Source: J Pers Med. 2022 Feb 8;12(2):236. doi: 10.3390/jpm12020236 (PMC8879244; doi:10.3390/jpm12020236)
Supplement: Supplementary file 1 [file jpm-12-00236-s001.zip › jpm-1474498-supplementary.pdf]

Table S1. Comparison of output and validation cohorts.

Parameters	Derivation cohort (n=406)	Validation cohort (n=333)	p-value
**Clinical parameters**			
Age [years]	53.62 ± 13.64	49.88 ± 10.71	**<0.0001**
Male [n (%)]	330 (81.3%)	264 (79.3%)	0.50
Symptoms duration [months]	40.13 ± 58.31	8.84 ± 15.84	**<0.0001**
BMI [kg/m2]	27.66 ± 5.11	29.41 ± 15.01	**0.04**
NYHA class	2.51 ± 0.89	2.23 ± 0.77	**<0.0001**
NYHA III/IV	194 (47.8%)	243 (73%)	**<0.0001**
Diabetes mellitus [n (%)]	90 (22.2%)	64 (19.2%)	0.33
Prior stroke [n (%)]	24 (5.9%)	13 (3.9%)	0.21
Liver diseases [n (%)]	53 (13%)	47 (14.1%)	0.68
Dyslipidemia [n (%)]	274 (67.5%)	262 (78.7%)	**0.0007**
COPD [n (%)]	27 (6.7%)	19 (5.7%)	0.60
Atrial fibrillation [n (%)]	129 (31.8%)	101 (30.3%)	0.67
**ECG findings**			
HR [bpm]	81.02 ± 20.3	76.5 ± 16.62	**0.007**
LBBB [n (%)]	105 (25.9%)	75 (22.5%)	0.29
VT [0/1]	106 (26.2%)	89 (26.6%)	0.89
**Echocardiographic findings**			
LVEF [%]	26.1 ± 9.37	27.02 ± 9.96	0.19
LVEDd [mm]	66.18 ± 10.39	65.08 ± 8.89	0.25
IVS [mm]	10.35 ± 2.18	10 ± 1.98	0.11
LAA [cm2]	29.42 ± 8.42	28.97 ± 8.33	0.45
RAA [cm2]	23.13 ± 8.2	22.2 ± 7.89	0.13
MR moderate/severe [n (%)]	192 (47.3%)	114 (34.2%)	**0.0003**
TR moderate/severe [n (%)]	105 (25.9%)	66 (19.8%)	0.05
**Laboratory results**			
Hb [g/dl]	14.27 ± 1.59	14.55 ± 1.66	**0.003**
eGFR [ml/min/1,73m2]	80.01 ± 21.48	83.5 ± 20.9	**0.01**
NT-proBNP [pg/ml]	3662.8 ± 7616.6	2759.3 ± 3639.6	**0.03**
LDL [mmol/l]	2.95 ± 0.98	2.99 ± 0.98	0.51
**HF therapy**			
BB [n (%)]	394 (97%)	321 (96.4%)	0.62
ACEi/ARB/ARNI [n (%)]	370 (91.1%)	294 (88.3%)	0.20
MRA [n (%)]	357 (87.9%)	289 (86.8%)	0.64
Furosemide [mg/d]	6.38 ± 19.6	6.26 ± 12.02	**<0.0001**
Loop diuretics [mg/d]	0.09 ± 0.28	0.16 ± 0.37	**<0.0001**
CRT [n (%)]	13 (3.2%)	11 (3.3%)	0.93
ICD [n (%)]	39 (9.6%)	30 (9%)	0.78
FU [months]	48.21 ± 31.97	41.63 ± 29.27	**0.01**
Deaths [n (%)]	70 (17.2%)	50 (14.9%)	0.39

Abbreviations: see Table 1.

Table S2. Comparison of the baseline characteristics of patients with high, and non-high, mortality risks calculated as the 2-year mortality risk according to the Krakow DCM Risk Score > or ≤ 6%.

Parameters	Q1-Q3 (n=529)	Q4 (n=176)	p-value
**Clinical parameters**			

Age [years]	51.31 ± 12.36	53.07 ± 13.46	**0.04**
Male [n (%)]	425 (80.34%)	143 (81.25%)	0.78
Symptoms' duration [months]	21.18 ± 36.83	43.19 ± 68.21	**<0.0001**
BMI [kg/m2]	28.95 ± 12.23	26.72 ± 5.21	**0.0004**
NYHA class	2.23 ± 0.78	2.85 ± 0.88	**<0.0001**
NYHA III/IV	183 (34.59%)	106 (60.23%)	**<0.0001**
Diabetes mellitus [n (%)]	89 (16.82%)	53 (30.11%)	**0.0001**
Prior stroke [n (%)]	11 (2.08%)	23 (13.07%)	**<0.0001**
Liver diseases [n (%)]	52 (9.83%)	41 (23.3%)	**<0.0001**
Dyslipidemia [n (%)]	411 (77.69%)	97 (55.11%)	**<0.0001**
COPD [n (%)]	36 (6.81%)	9 (5.11%)	0.38
Atrial fibrillation [n (%)]	149 (28.17%)	68 (38.64%)	**0.009**
**ECG findings**			
HR [bpm]	77.66 ± 17.92	83.6 ± 21.39	**0.0003**
LBBB [n (%)]	106 (20.04%)	68 (38.64%)	**<0.0001**
**Echocardiographic findings**			
LVEF [%]	27.24 ± 9.45	23.67 ± 9.49	**<0.0001**
LVEDd [mm]	65.06 ± 8.59	68.42 ± 12.32	**0.0007**
IVS [mm]	10.28 ± 2.1	9.93 ± 2.15	**0.03**
LAA [cm2]	28.24 ± 7.68	32.15 ± 9.79	**<0.0001**
RAA [cm2]	21.85 ± 7.11	25.32 ± 10.12	**<0.0001**
MR moderate/severe [n (%)]	194 (36.67%)	100 (56.82%)	**<0.0001**
TR moderate/severe [n (%)]	89 (16.82%)	74 (42.05%)	**<0.0001**
**Laboratory results**			
Hb [g/dl]	14.74 ± 1.46	13.39 ± 1.63	**<0.0001**
eGFR [ml/min/1,73m2]	83.68 ± 18.1	76.04 ± 26.8	**<0.0001**
NT-proBNP [pg/ml]	1963.62 ± 2478.54	7091.83 ± 10736.67	**<0.0001**
Cholesterol LDL [mmol/l]	3.09 ± 0.96	2.55 ± 0.92	**<0.0001**
**HF therapy**			
BB [n (%)]	516 (97.54%)	167 (94.89%)	0.09
ACEi/ARB/ARNI [n (%)]	503 (95.09%)	131 (74.43%)	**<0.0001**
MRA [n (%)]	461 (87.15%)	160 (90.91%)	0.21
Furosemide [mg/d]	25.5 ± 40.68	69.89 ± 83.54	**<0.0001**
Loop diuretics [mg/d]	40.62 ± 61.69	101.87 ± 132.92	**<0.0001**
CRT [n (%)]	4 (0.76%)	18 (10.23%)	**<0.0001**
ICD [n (%)]	41 (7.75%)	27 (15.34%)	**0.003**
**Follow-up**			
FU [months]	47.25 ± 31.2	39.3 ± 30.6	**0.002**
Deaths [n (%)]	44 (8.32%)	75 (42.61%)	**<0.0001**

Abbreviations: see Table 1.

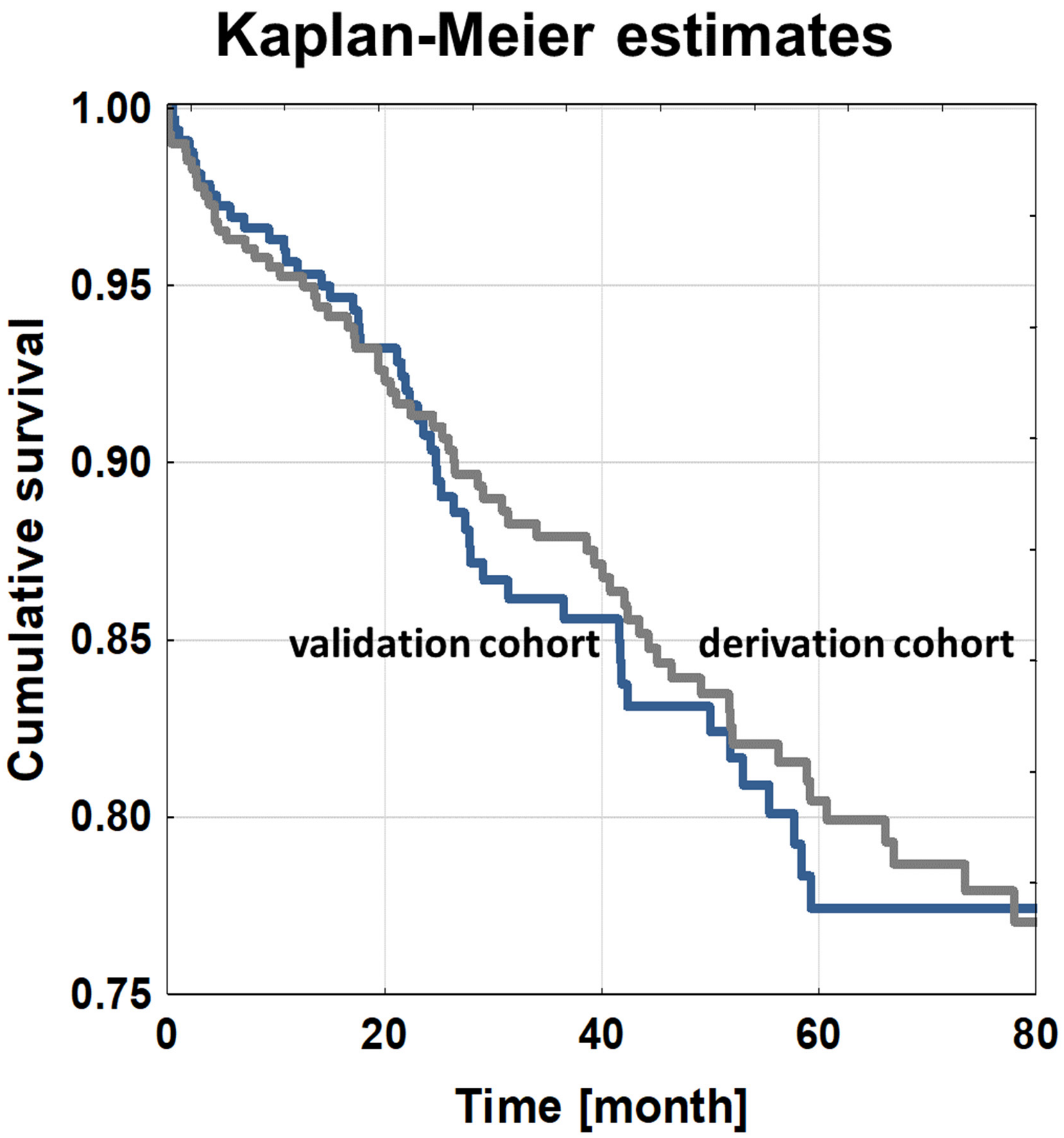

Figure S1. Comparison of mortality rate between derivation and validation cohorts based on Kaplan-Meier estimates.
